# A time-efficient web-based teaching tool to improve medical knowledge and decrease ABIM failure rate in select residents

**DOI:** 10.3402/meo.v20.29221

**Published:** 2015-10-30

**Authors:** Sean M. Drake, Waqas Qureshi, William Morse, Kimberly Baker-Genaw

**Affiliations:** 1 Department of Internal Medicine, Henry Ford Hospital, Detroit, MI, USA; 2 Division of Cardiovascular Medicine, Wake Forest School of Medicine, Wake Forest University, Winston Salem, NC, USA

**Keywords:** teaching tool, web-based, In-Training Examination, internal medicine residency, residents, teaching physicians

## Abstract

**Aim:**

The American Board of Internal Medicine (ABIM) exam's pass rate is considered a quality measure of a residency program, yet few interventions have shown benefit in reducing the failure rate. We developed a web-based Directed Reading (DR) program with an aim to increase medical knowledge and reduce ABIM exam failure rate.

**Methods:**

Internal medicine residents at our academic medical center with In-Training Examination (ITE) scores ≤35th percentile from 2007 to 2013 were enrolled in DR. The program matches residents to reading assignments based on their own ITE-failed educational objectives and provides direct electronic feedback from their teaching physicians. ABIM exam pass rates were analyzed across various groups between 2002 and 2013 to examine the effect of the DR program on residents with ITE scores ≤35 percentile pre- (2002–2006) and post-intervention (2007–2013). A time commitment survey was also given to physicians and DR residents at the end of the study.

**Results:**

Residents who never scored ≤35 percentile on ITE were the most likely to pass the ABIM exam on first attempt regardless of time period. For those who ever scored ≤35 percentile on ITE, 91.9% of residents who participated in DR passed the ABIM exam on first attempt vs 85.2% of their counterparts pre-intervention (*p*<0.001). This showed an improvement in ABIM exam pass rate for this subset of residents after introduction of the DR program. The time survey showed that faculty used an average of 40±18 min per week to participate in DR and residents required an average of 25 min to search/read about the objective and 20 min to write a response.

**Conclusions:**

Although residents who ever scored ≤35 percentile on ITE were more likely to fail ABIM exam on first attempt, those who participated in the DR program were less likely to fail than the historical control counterparts. The web-based teaching method required little time commitment by faculty.

In-Training Examinations (ITEs) are used in residency training programs as an objective measure to assess a learner's medical knowledge base (1). For internal medicine, the test was designed and is administered by the American College of Physicians along with support from the Association of Program Directors in Internal Medicine and the Association of Professors in Medicine (2). The ITE model was originally based on the construct used for board certification examinations (3). Previous research has demonstrated the predictive value of ITE results, with reported correlations between ITE and subsequent written boards in various specialties, including internal medicine (4, 5).

Use of ITE scores to develop educational interventions has not been well studied. Moreover, interventions to improve ITE scores have shown conflicting results. Types of these interventions include educational conferences (6–8), individualized study plans (9–11), frequent meetings with faculty (9, 11, 12), online cases (12), practice questions (10, 12–15), and group learning (16). All of these methodologies require significant time commitment by teaching physicians.

Considering the high stakes for residents and residency programs for passing the board examination and critical need for core knowledge development for clinical application, we, at the Henry Ford Hospital Internal Medicine residency developed a web-based teaching and feedback tool called the Directed Reading (DR) program for our internal medicine residents. We hypothesized that this tool would facilitate self-directed learning and increase medical knowledge, improve the ITE scores of internal medicine residents, and reduce the American Board of Internal Medicine (ABIM) exam failure rates in our graduates.

## Methods

### Study participants

The internal medicine residency program at our large, urban, teaching hospital includes a total of 120 residents. Residents who obtained ≤35th percentile on the ITE from 2007 to 2013 were enrolled in the DR program. Residents with ≤35th percentile on the ITE from 2002 to 2006 were used as a historical control group for analyses. We chose to use this cutoff percentile, rather than percentage, to be able to compare our results with previous studies that showed high likelihood of failure on the ABIM exam for residents scoring ≤35th percentile on ITE (4, 5).

Our internal medicine residency policy requires all residents to take the ITE. For the study period, residents took the ITE in October and those with scores ≤35th percentile received in December then started the DR program in January. Residents could be enrolled in DR more than one year depending on subsequent ITE scores. This study was approved by the Institutional Review Board at Henry Ford Hospital.

### Directed Reading program

The DR program was developed in 2007 as an ongoing formative assessment for residents by one of the authors (WM) and is available to the general public as an open source software package. The main aim of the DR program was to provide a web-based platform for residents and faculty to mutually review and provide feedback regarding the educational objectives provided by the American College of Physicians based on each resident's performance on the ITE.

**Fig. 1:**
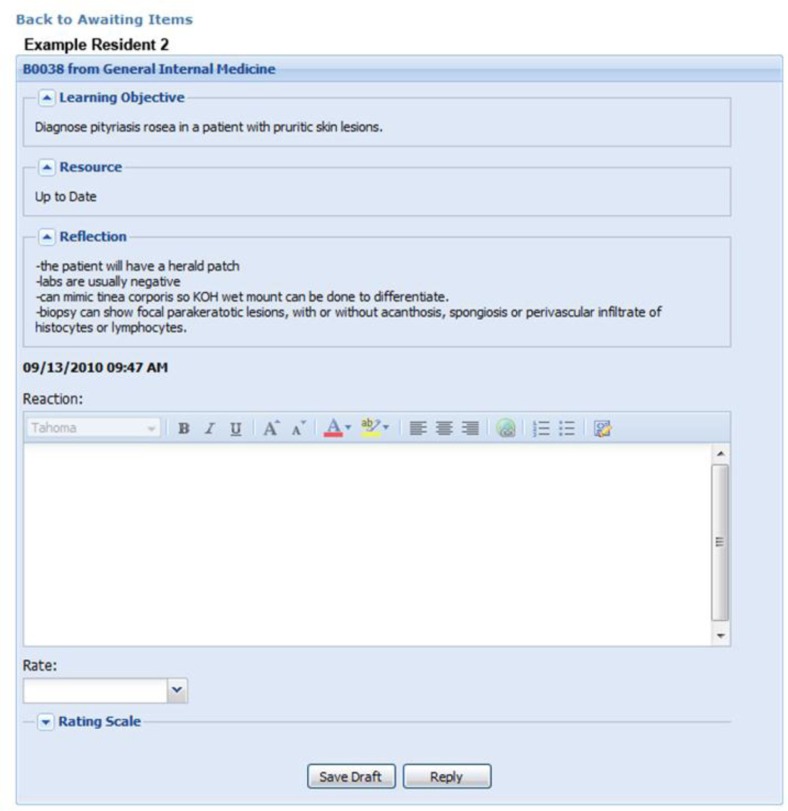
Screenshot of resident and teaching physician Directed Reading web pages.

For residents enrolled in the DR program from 2007 to 2013, ITE educational objectives answered incorrectly by each resident were obtained and loaded onto that resident's personalized DR web page. Residents were encouraged to use standard references to read on their identified objectives, including online textbooks and peer-reviewed literature. As shown in an example of an assignment in Fig. 1, the ‘learning objective’ matches an educational objective answered incorrectly on the ITE, ‘resource’ indicates the source of the information read by the resident (such as Up to Date, DynaMed, Medical Knowledge Self-Assessment Program, a textbook, or a primary journal article), and the ‘reflection’ is the resident's written response to demonstrate understanding of that educational objective. When completed, the website application sends an email to the resident's DR preceptor who reviews and rates the resident's answer as either satisfactorily completed or unsatisfactory. If unsatisfactory, the preceptor writes a brief response (‘reaction’ in Fig. 1) which is sent to the resident. When revision is required, the educational objective maintains an unfinished status with preceptor comments and remains available for the resident to revise and resubmit.

DR program residents were expected to complete five learning objectives weekly. DR residents started DR in January and continued until all their assigned ITE educational objectives were completed. Total number of educational objectives ranged from 122 to 180, which required about 24–36 weeks based on five objectives completed per week.

DR preceptors were assigned four or five residents each. To provide additional oversight, a weekly report was generated from the DR website data, including number of completed submissions per resident and number of pending evaluations for preceptor review. Reports were reviewed by the program director (PD) every week during the faculty meeting. The DR preceptors included the PD, associate program directors (APDs), and the clerkship director, all of whom are board-certified internists.

### Data analysis

Data analysis aimed at determining the ABIM exam pass rate before and after DR implementation and whether this rate changed over time among those who were and were not eligible for DR. Rates were compared using chi-squared tests or Fisher's exact tests. To compare the ABIM exam pass rates across time and eligibility groups, pairwise comparisons were performed and the Benjamini–Hochberg adjustment (17) was applied to the *p*-value to control the false discovery rate. In addition, a univariate repeated measures model was used to examine the effect of time period (pre-intervention 2002–2006 vs post-intervention 2007–2013) on ITE percentile.

### DR time survey

At the end of the study in 2013, DR preceptors (*n*=7) and residents enrolled in the DR program at that time (*n*=26) were asked to complete a survey about the time commitment required for the DR program. Participation was voluntary. The responses were graded on a five-point Likert scale (5=strongly agree, 1=strongly disagree).

## Results

The pre-intervention group (2002–2006) included 181 residents and the post-intervention group (2007–2013) 263 residents. Using an ITE score ≤35 percentile to qualify for the DR intervention, 81 residents qualified for DR in the pre-intervention period and 111 residents qualified for and participated in DR in the post-intervention period. The DR software administrative reports showed that 97% of the 111 DR participants completed at least 90% of assigned objectives.

Comparison groups across the full time period of the study are shown in Table 1. Residents who never scored ≤35 percentile on ITE were significantly more likely to pass the ABIM exam on first attempt compared to residents who ever scored ≤35 percentile on ITE across the full time period (99.2% vs 89.1%, *p<*0.001). This finding was also observed in the pre-intervention period (100% vs 85.2%, *p<*0.001), but no significance was found in the post-intervention period (98.7% vs 91.9%, *p=*0.010). For those who ever scored ≤35 percentile on ITE, 91.9% of residents who participated in DR passed the ABIM exam on first attempt compared to only 85.2% of their control counterparts (*p*<0.001; Table 2).

**Table 1:** Resident ITE scores 2002–2013 (*N*=446)

Group	Residents with ITE ≤35%	Residents with ITE >35%
ITE first attempt	120 (26.9%)	326 (73.1%)
ITE second attempt	122 (27.4%)	324 (72.7%)
ITE third attempt	98 (22%)	348 (78%)
Ever scored	192 (43.1%)	254 (57%)
Total times		
Never	254 (57.0%)	
1	92 (20.6%)	
2	52 (11.7%)	
3	48 (10.8%)	

**Table 2:** ABIM exam pass/fail first attempt and DR eligibility over time

Pass first attempt	Never ≤35%, pre-intervention	Never ≤35%, post-intervention	Ever ≤35%, pre-intervention	Ever ≤35%, post-intervention	*P*
No	0	2 (1.3%)	12 (14.8%)	9 (8.1%)	<0.001
Yes	100 (100%)	162 (98.7%)	69 (85.2%)	102 (91.9%)	

A comparison of ITE scores by time period showed that the adjusted mean ITE percentile was significantly higher in the post-intervention group (54.4% pre- vs 59.1% post-intervention, *p=*0.0017).

Regarding the survey for feedback on the DR intervention, six of seven teaching physicians (85%) responded. The average time required by teaching physicians to search and read about the DR objective was 7 min (range 4–10 min). The average time to provide feedback in written format to the resident was 3 min (range 2–6 min). The survey response rate for DR residents was 96% (25 of 26). The average time required by residents to search and read about the DR objective was 25 min (range 10–50 min). The average time to provide response in written format by the resident was 20 min (range 5–75 min).

## Discussion

This study demonstrated that the DR intervention was associated with an increase in ABIM exam first-attempt pass rate in the subset of residents who scored ≤35th percentile on the ITE. Those residents who participated in DR had a 6.7% absolute increase in ABIM exam pass rate compared to historical counterparts. In addition, the ≤35 percentile ITE group that participated in the DR intervention showed significantly higher ITE scores than their historical counterparts. This emphasizes the need to identify low-scoring ITE residents early and enroll them in DR sooner, as our study also showed that residents who ever scored above the 35th percentile on ITE had a significantly greater likelihood of passing the ABIM exam on first attempt.

**Fig. 2:**
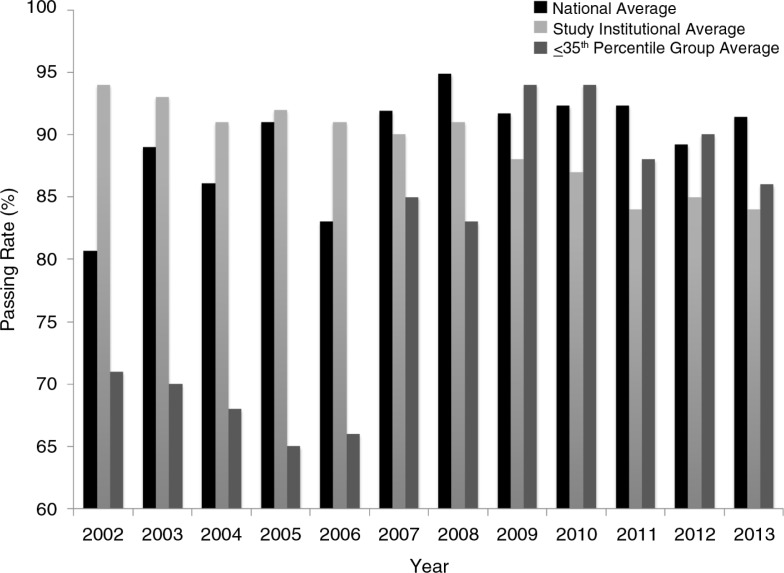
Time trend of American Board of Internal Medicine (ABIM) failure rates nationally and at the institution. Directed Reading was instituted in 2007. Note the increase in passing rate; a slight decrease occurred after 2010 which is consistent with national decrease in passing rates.

This supports findings from Waxman and others that residents scoring ≤35 percentile on ITE are more likely to fail the ABIM examination (5). An unexpected finding was that our institution's ABIM exam pass rate has remained higher despite a declining trend nationally (Fig. 2). Although the cause of this may be multifactorial, one reason may be use of the DR program.

Our study provides a novel method of utilizing web-based tools to monitor, provide feedback, and foster independent learning with a short time commitment. We believe that the success of the DR program is its direction, oversight, and guidance to read about areas of personal weakness as demonstrated on the ITE. The web-based tool not only provides a way to track residents’ progress but also offers a platform for individual faculty development.

Significant improvement occurred in ITE scores in the post-intervention group. Although the DR program may be a factor in improved ITE scores, there may be other factors as well. However, this finding is consistent with a recent study validating an equation for predicting probability of passing the ABIM exam (18). This predictive equation relies heavily on ITE scores and hence improvement in ITE scores may in turn result in improvement in odds to pass the ABIM exam.

Our study has several strengths. First, this is a relatively large sample size with six years of longitudinal data. Second, the study demonstrated a significant improvement in ABIM exam pass rates in select residents, even though the national trend is in the opposite direction, which further strengthens the value of this program. Third, we included the time commitment which provides important practical information about the utility of this program. With each faculty assigned only a few residents and each resident completing five DR objectives per week, the time commitment is very manageable (~1 h or less per week). In addition, the web-based feedback approach removes the often negative effects of face-to-face confrontation and cultivates an environment of mentorship and collaborative learning. The DR program capitalizes on asynchronous learning and feedback, allowing both the learner and faculty to review the objectives at times convenient for each, and not constraining either party. The DR software, which is available free of charge, is easily modifiable to any discipline and may be personalized for any learning objective.

This study also has limitations. We utilized the retrospective publically available data on ABIM exam failure rates of the institution but did not collect information about learning resources used by non-DR residents. However, a prior study that reported increased use of electronic resources by residents (6) showed subsequent improvement in ITE. Our residents did rely heavily upon electronic resources. Another limitation is that this was a single site study. Our faculty physicians are employed in a large group practice and have protected time for program administration, which may affect their participation rates. There might be differences in familiarity with computers among our residents and teaching physicians which were not studied. However, the DR website was easy to navigate and was constructed similar to a general email inbox.

Our study provides significant evidence to suggest that DR was associated with improved ABIM exam pass rates in residents who scored ≤35 percentile on the ITE compared to historical controls. The program was well received by residents as well as the teaching physicians with minimal time commitment by the latter. Further research is needed in larger multi-site studies to evaluate the impact of such programs, especially with the recent implementation of the ACGME Next Accreditation System.
